# Exploring the young demographic profile of COVID-19 cases in Hong Kong: Evidence from migration and travel history data

**DOI:** 10.1371/journal.pone.0235306

**Published:** 2020-06-26

**Authors:** Christian Joy Pattawi Cruz, Rachel Ganly, Zilin Li, Stuart Gietel-Basten

**Affiliations:** 1 Division of Public Policy, The Hong Kong University of Science and Technology, Kowloon, Hong Kong SAR, People’s Republic of China; 2 Division of Social Science, The Hong Kong University of Science and Technology, Kowloon, Hong Kong SAR, People’s Republic of China; University of West London, UNITED KINGDOM

## Abstract

This paper investigates the profile of COVID-19 cases in Hong Kong, highlighting the unique age structure of confirmed cases compared to other territories. While the majority of cases in most territories around the world have fitted an older age profile, our analysis shows that positive cases in Hong Kong have been concentrated among younger age groups, with the largest incidence of cases reported in the 15–24 age group. This is despite the population’s rapidly aging structure and extremely high levels of population density. Using detailed case data from Hong Kong’s Centre for Health Department and Immigration Department, we analyze the sex and age distribution of the confirmed cases along with their recent travel histories and immigration flows for the period January to April 2020. Our analysis highlights Hong Kong’s high proportion of imported cases and large overseas student population in developing COVID-19 hotspot areas such as the United Kingdom. Combined with community action and targeted and aggressive early policy measures taken to contain the virus, these factors may have contributed to the uniquely younger age structure of COVID-19 cases in the city. Consequently, this young profile of confirmed cases may have prevented fatalities in the territory. Recent research has highlighted the importance of a demographic approach to understanding COVID-19 transmission and fatality rates. The experience in Hong Kong shows that while an older population age structure may be important for understanding COVID-19 fatality, it is not a given. From a social science perspective at least, there is ‘no easy answer’ to why one area should experience COVID-19 differently from another.

## Introduction

The Hong Kong Special Administrative Region of the People's Republic of China (hereafter Hong Kong) is a city and special administrative region of China in the eastern Pearl River Delta by the South China Sea. In 2019, Hong Kong had roughly 7.5 million people in a 1,104-square-kilometre (426 square miles) territory while in Kowloon, where more than three in ten residents reside, population density is at 48,930 persons per square kilometer. This makes it one of the most densely populated territories in the world [1]. Apart from being a rapidly aging society [2,3], Hong Kong is also a migration destination. For the period 2015 to 2020, the net migration number was 147,000 or a net of four in-migrants per 1,000 of the population [1].

Hong Kong faces the challenge of infectious diseases as a consequence of various factors, including high population density, increasing environmental pollution, high migration inflows and outflows, the emergence of new infections as well as the changing lifestyle and behavior of its residents [4,5]. The most recent significant outbreak was the severe acute respiratory syndrome (SARS) outbreak which reached Hong Kong in March 2003 [5]. Based on World Health Organization (WHO) data until July 11, 2003, a total of 1,755 SARS cases had been identified in the territory, of which 298 people died of the disease [6]. At that point, it was largely believed that Hong Kong was unprepared to be one of the epicenters of the SARS epidemic. From this recent experience, did Hong Kong learn from the hard lessons of the past?

The WHO officially declared the novel Coronavirus infections (hereafter COVID-19) outbreak as a pandemic on March 11, 2020, after it had spread to more than 100 countries and resulted in tens of thousands of cases within a few months. In Hong Kong, however, the first case was reported on January 23, 2020. In early February, the government was strongly criticized for policy responses related to a variety of issues, including the legality of, and access to face masks; border closure; medical fees and quarantine policy [7]. During this early period, unfavorable comparisons were made with other regional governments (especially Macau and Singapore) who appeared to be managing the crisis more effectively [8,9]. However, by the time of writing in June 2020, new cases being reported in Hong Kong–especially local transmissions—are very rare [10]. That this has occurred without the general lockdown policies seen in other parts of the world is even more remarkable.

Our study includes an examination of the age and sex distribution of the COVID-19 confirmed cases in Hong Kong and an exploration of how the different measures to combat this outbreak resulted in a relatively low number of cases and deaths. Specifically, as demographers, we wished to explore the extent to which insights from demographic science could assist in explaining the nature of the Hong Kong experience of COVID-19. In this paper, we highlight the potential impact of the young profile of the confirmed cases on the total number of mortalities and the effect of early, aggressive policy measures including travel bans, enforced quarantines and contact-tracing imposed by the Hong Kong government as early as January 27, 2020 in containing the spread of the COVID-19.

## Materials and methods

Data on confirmed COVID-19 cases were taken from the Centre for Health Protection (CHP) of the Hong Kong Department of Health. We assessed the age and sex distribution of the confirmed cases, discharge status (discharged, hospitalized or died) and type of transmission. We obtained the data regarding cumulative cases by age group from January 23 to April 16, 2020, which allowed us to determine the age group that registers the highest number of confirmed cases over time. Daily migrant inflows and outflows data was retrieved from the Hong Kong Immigration Department showing arrivals at each border checkpoint into Hong Kong broken down by citizenship status. In addition, we retrieved detailed archived datasets pertaining to the travel histories of confirmed COVID-19 cases, which are updated on an almost daily basis by the CHP. We excluded travel histories pertaining to domestic travel (buses, trains and ferries) and a small number of journeys pertaining to outbound from Hong Kong. We linked travel histories to confirmed case identifications (IDs) in order to examine the age structure and timing of cases where the apparent source of infection was not in Hong Kong (i.e. not a community infection). These include cases with a travel history from countries with widespread infection or where infection from a confirmed case who traveled occurred. By doing so, we highlight the importance of returnee Hong Kong residents from overseas hotspots on the relatively young age structure of confirmed cases during the second wave in March 2020. We also gathered data on policy measures implemented by the Hong Kong government in order to highlight the possible impact of major border closures and quarantine arrangements imposed by the Hong Kong government in reducing further numbers of imported COVID-19 infections. We also utilized secondary data for comparative demographic analyses from the Hong Kong Census and Statistics Department, United Nations Population Division and the Chinese Center for Disease Control and Prevention.

## Results

### Age profile of confirmed COVID-19 cases

**Fig 1:**
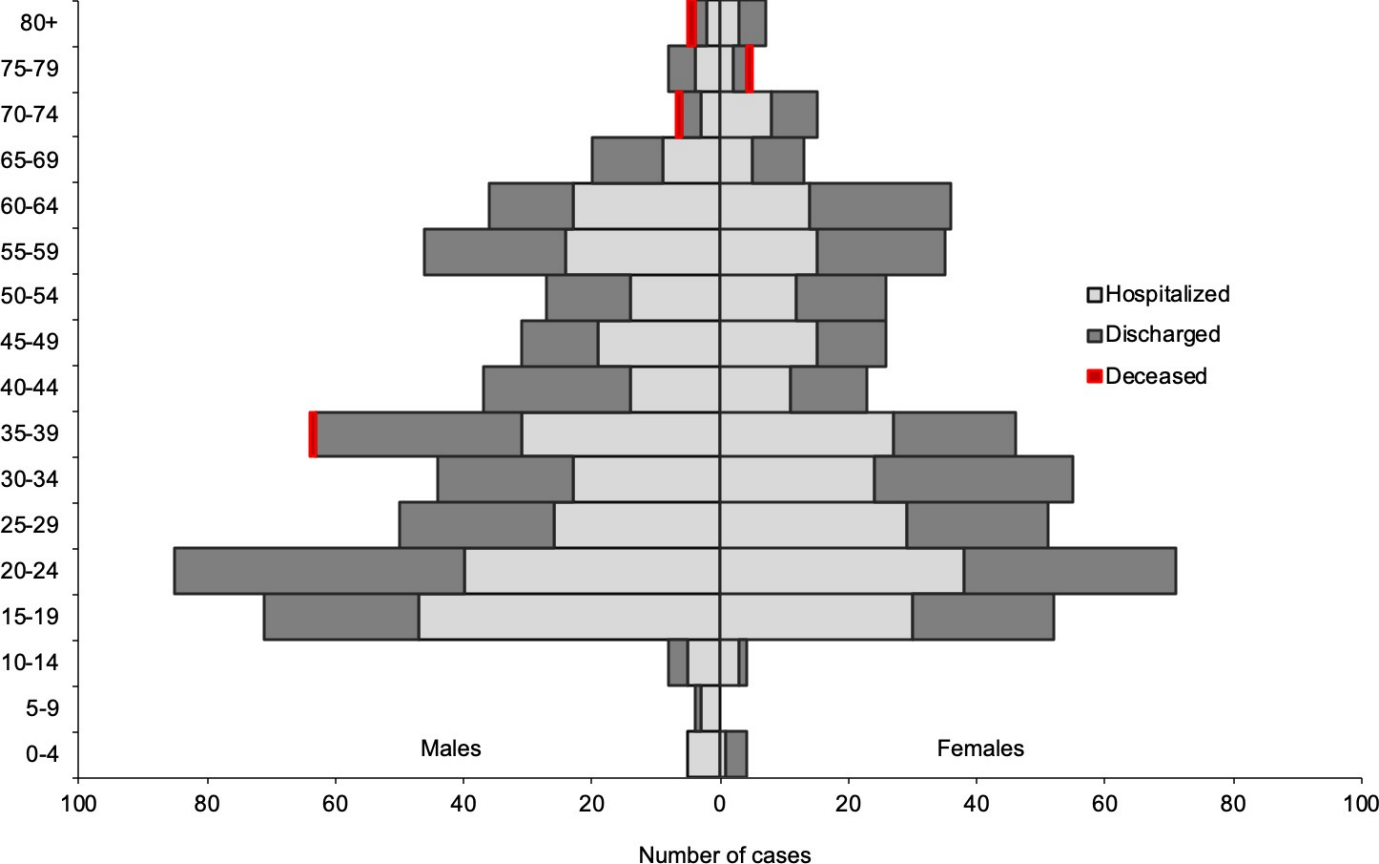
Age and sex distribution of confirmed COVID-19 cases Hong Kong (n = 1,017, as of April 16, 2020). Data Source: Hong Kong Center for Health Protection, Department of Health.

Compared with the aging Hong Kong population [1], the COVID-19 confirmed cases have an entirely different distribution. Fig 1 shows the age and sex distribution of the 1,017 confirmed COVID-19 cases in Hong Kong as of April 16, 2020. In Hong Kong, the majority (63.8%) of confirmed cases are in the 15 to 44 age bracket. Broken down further, 27.4% of cases are aged 15–24, 19.7% aged 25–34 and 16.7% aged 35–44. Less than a tenth (7.9%) are aged 65 and over. This age distribution of the local COVID-19 confirmed cases does not fit the general profile of other territories wherein the infections are more concentrated among those in the older age groups. Although, of course, this may be related to testing patterns by age, rather than true differences in incidence. The *WHO Coronavirus Disease 2019 (COVID-19) Situation Report 89* shows that as of April 13, 2020, there were a total of 716,570 confirmed cases with reported information on age and sex coming from 113 countries, territories and areas; of these confirmed cases, the median age for males is 52 (interquartile range, IQR: 35–64) years and for females 50 (IQR 35–64) years [11]. Examples of territories with an older profile of confirmed cases are Mainland China where the majority of confirmed cases (54.0%) as of February 11, 2020 belong to the older age groups, at least 50 years old [12], and the Philippines wherein as of March 25, 2020, the majority of the confirmed cases are in the age group 50 and older [13].

There is also an imbalanced sex ratio among the COVID-19 confirmed cases in Hong Kong with a higher proportion of male cases, representing 54% of the total. The number of male cases outnumbered females in almost all age groups, except in the 25–34 age group where they made up only 47% of cases. For all other age groups, male cases were between 50 to 60% of the total; an exception was for those aged under 15 of which 68% of cases were male, however, the total number of cases at this age was extremely small (25 out of the total 1,017 cases). This sex profile with a higher prevalence of male cases is similar to the outbreaks in other territories [12,13] The youngest confirmed cases in Hong Kong were two males under one-year-old while the oldest confirmed case is a 96-year-old female. Nearly half of the 1,017 confirmed cases (48.0%) had already been discharged from the hospital as of April 16, 2020.

In short, COVID-19 confirmed cases in Hong Kong were found primarily among the working and school-age groups, and to a certain degree, among men. By April 16, 2020, almost three months after the first reported case on January 23, 2020, four deaths among confirmed COVID-19 cases were reported in Hong Kong. Three such deaths were over 70 years old (two males and one female) while the fourth was the case of a 39-year old male. It is possible that this young age structure of confirmed cases in Hong Kong contributed to the extremely low numbers of fatalities compared to other territories.

**Fig 2:**
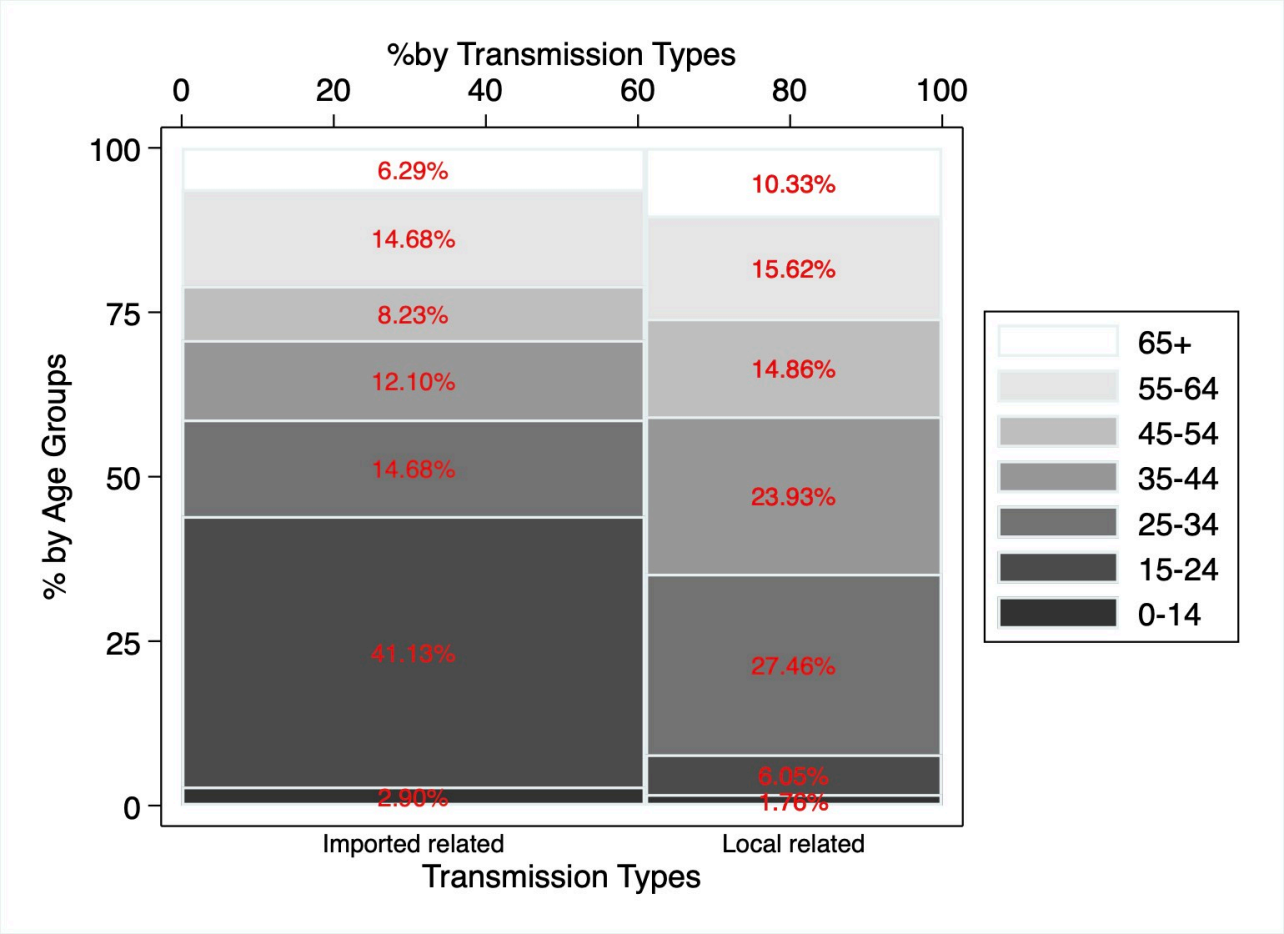
Distribution of confirmed COVID-19 cases by transmission type and by age groups: Hong Kong (n = 1,017, as of April 16, 2020). Data Source: Hong Kong Centre for Health Protection, Department of Health.

A large proportion of Hong Kong’s confirmed cases were ‘imported’. Three in five confirmed cases (61.0%) have travel history from countries with widespread infection or were directly infected by a confirmed case who travelled; hence, they are considered cases with imported transmission (see Fig 2). Two-fifths of these imported confirmed cases (41.1%) are young adults in the age group 15–24 years old. The proportion of imported versus local cases differs significantly based on age group. Nine in ten of the confirmed cases in the 15–24 age group (91.4%), the group with the largest incidence of COVID-19, were in fact imported. In contrast, 59.7% of cases aged 55–64 and less than half of those in other age groups are considered imported.

**Fig 3:**
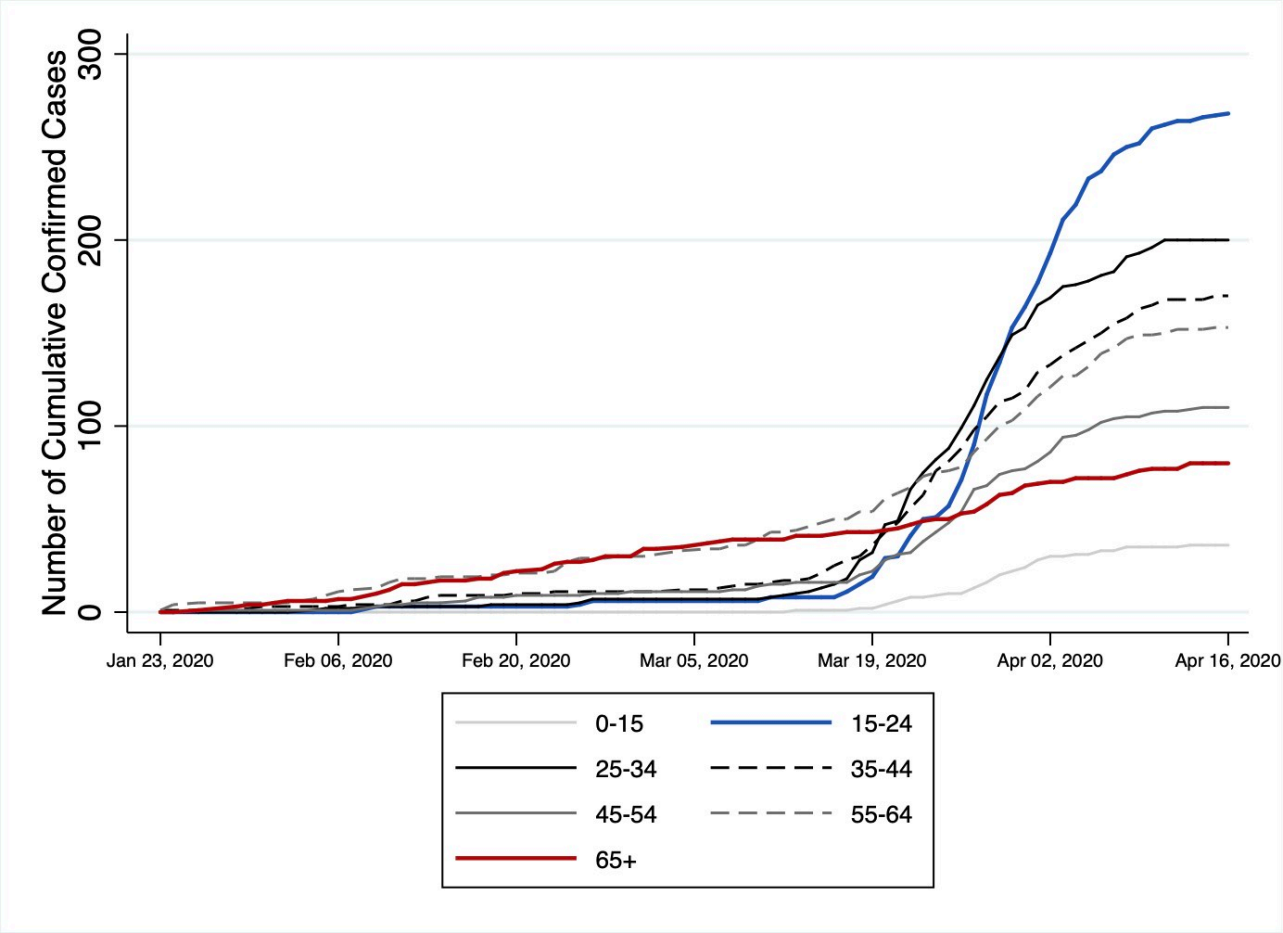
Number of cumulative confirmed cases by age groups: Hong Kong (n = 1,017, January 23 to April 16, 2020). Data Source: Hong Kong Center for Health Protection, Department of Health.

Most of Hong Kong’s cases in the younger age groups occurred in a second wave of infections during March. Before March 18, 2020, the age group with the highest frequency of COVID-19 cases was 55–64 followed closely by the 65 and over age group (see Fig 3). However, after March 18, 2020 the number of new cases in Hong Kong sharply increased, most of these being imported cases in younger age groups. This timing of these imported cases is similar to that experienced by China wherein the imported cases more than doubled starting from March 18, 2020, mostly coming from countries with high outbreak of COVID-19 infections like the United Kingdom (UK), United States of America (USA) and Spain [14]. However, age-specific data about imported cases is not available in China.

### Travel history of COVID-19 cases

Analysis of the travel histories of Hong Kong cases suggests that the majority of imported cases in the 15–24 age group may be Hong Kong residents who are studying or working abroad. The travel history data collected and archived by the CHP over the study period was matched to case numbers from the case data to study the travel history and age profile of all known imported cases. The data collected by the CHP contains the travel histories of all confirmed COVID-19 cases up to fourteen days before the case was reported and formed part of attempts of the government to track and trace the movements of all newly reported cases. This is done in order to contact and isolate anyone with whom they had close contact in the days before the onset of symptoms, including those whom they may have sat close to on flights. Of the 508 COVID-19 cases with an overseas travel history tracked by the CHP, 47.4% were imported from the UK, 9.1% from the USA and 3.9% each from Qatar, Canada and Switzerland. By far the largest age group with a travel history was those aged 15–24 with 205 cases, of which the majority were from the UK (62.4%), 8.3% from the USA, and 4.4% to 5.4% each from Switzerland, Qatar and the Netherlands. The next largest group with travel histories was those aged 25–34 with 86 cases, nearly half of these coming from the UK (48.8%). We are not able to ascertain the employment or other characteristics of these cases.

Hong Kong has a large population of mobile residents who hold citizenship or permanent residency but work, study or are retired overseas. According to the 2016 by-census, just under 220,000 permanent residents of Hong Kong were deemed ‘mobile’, meaning they had spent between one to three months in the city of the previous six [15]. In 2016, the largest population of mobile residents was those aged 15–24, with a total of 47,938 most of whom were registered as students. We were not able to obtain the country of temporary overseas residency from the census data. The next largest population of mobile residents were those aged 65 and over (42,299) who were mostly registered as retired, while a further 40,926 were aged 55 to 64 mostly employees or retirees. In addition, according to the 2016 by-census Hong Kong is home to a sizable number of non-permanent residents; many of these residents may return to their home countries on a regular basis for study, work, or family visits. The largest groups of non-Chinese nationals were Filipinos (186,000) and Indonesians (159,901), most of whom work as domestic workers on temporary foreign-worker permits. Hong Kong was also home to 35,069 British citizens, almost 15,000 Americans and 66,690 citizens from South Asian countries (India, Nepal and Pakistan), while a further 121,775 Hong Kong Chinese were registered as being domiciled overseas in 2016 [15].

**Fig 4:**
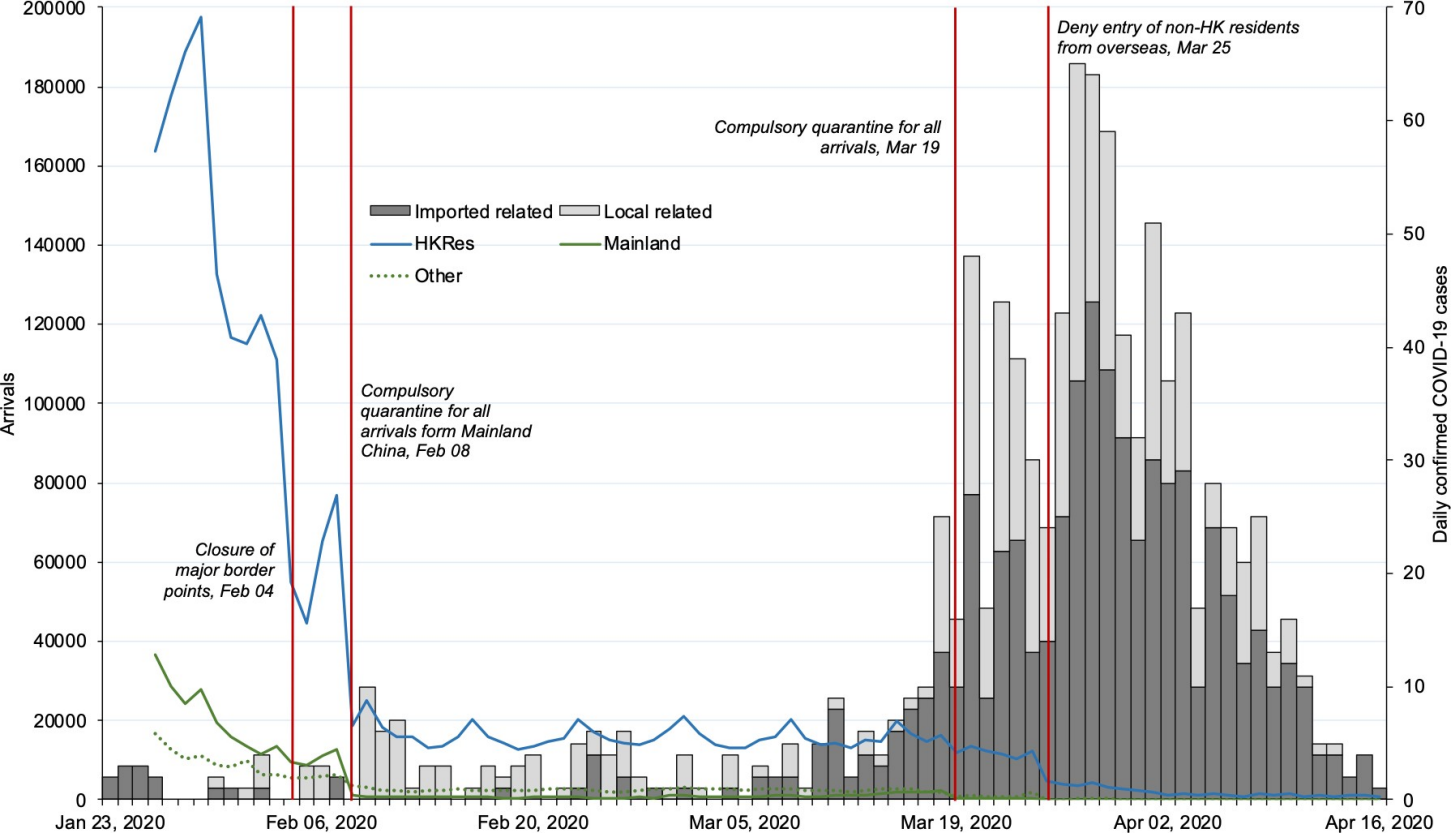
Number of arrivals (primary axis) and daily confirmed cases by residential status (secondary axis: Hong Kong (n = 1,017, January 23 to April 16, 2020 [arrivals data only available from January 24, 2020]). Data Source: Hong Kong Immigration Department; Hong Kong Center for Health Protection, Department of Health.

The large increases in newly confirmed cases in the younger age groups, the majority of which were imported, occurred from March 10, 2020 onward (Fig 4). This was in tandem with a large increase in confirmed cases and deaths throughout multiple countries in Europe, USA and Canada. On March 13, 2020, the Hong Kong Security Bureau had announced a ‘Red Outbound Travel Alert’ (‘Red OTA’) for the Schengen area, announcing a mandatory 14-day home quarantine for all arrivals from the Schengen area. This was followed by a ‘Red OTA’ for Ireland, United Kingdom and the USA on March 15, 2020 with the announcement of home quarantine arrangements for all travelers from the three states to begin four days later (see S1 Table for a full timeline of policy events).

Fig 4 shows the arrivals into Hong Kong over the period from January 24 to April 16, 2020. Most arrivals were Hong Kong residents travelling via the airport. On February 4, 2020, all land and sea border points were closed except for two control points–the Shenzhen Bay control point and Hong Kong-Zhuhai-Macao Bridge. Daily arrivals into the city fell dramatically after the end of the Chinese Lunar New Year holidays at the end of January 2020, and took another dramatic fall after the home quarantine arrangements for all arrivals from China were put into place on February 8, 2020. Arrival numbers followed a relatively steady pattern at under 25,000 per day until dropping off sharply from March 19, 2020, when the new 14-day home quarantine arrangements were put in place for all arrivals into the city, regardless of whether or not they were residents. From March 25, 2020, all non-residents were barred from entering the city except for nationals of Macau, Taiwan or mainland China.

### Other measures taken by the Hong Kong government

These border closures and sharply lower inbound-travel movements together with Hong Kong’s aggressive policy of testing, contact-tracing and quarantine of confirmed cases and their close contacts (see S1 Table) undoubtedly contributed to the sharp decline in newly confirmed cases during the month of April [16]. On April 20, 2020, Hong Kong recorded its first day without a confirmed case, from a peak of more than 60 cases per day in late March. Social distancing and rapid population behavioral changes also likely played a role, with measures such as wearing masks, working from home and school closures leading to an estimated 44.0% reduction in seasonal influenza incidence [16]. The containment of a severe local outbreak of COVID-19 in Hong Kong thus far, and the very high incidence of confirmed cases in younger age groups mostly among Hong Kong residents returning to the city from overseas hotspot areas, have surely contributed to the very low number of fatalities in the city-state, with only four deaths reported by April 23, 2020 out of 1,030 confirmed cases. One death occurred in the 80 years and over age group, out of twelve total cases. In Italy, China and South Korea, vastly higher case fatality rates were recorded for those in the older age groups; as at March 31, 2020 case fatality rates for those above 80 years old were at 27.7% in Italy and 18.3% in South Korea [17,18].

## Discussion

This paper takes a social scientific approach to illustrate the particularities of COVID-19 outcomes in Hong Kong up until now. The paper is a simple, descriptive analysis of the available data on hand.

**Fig 5:**
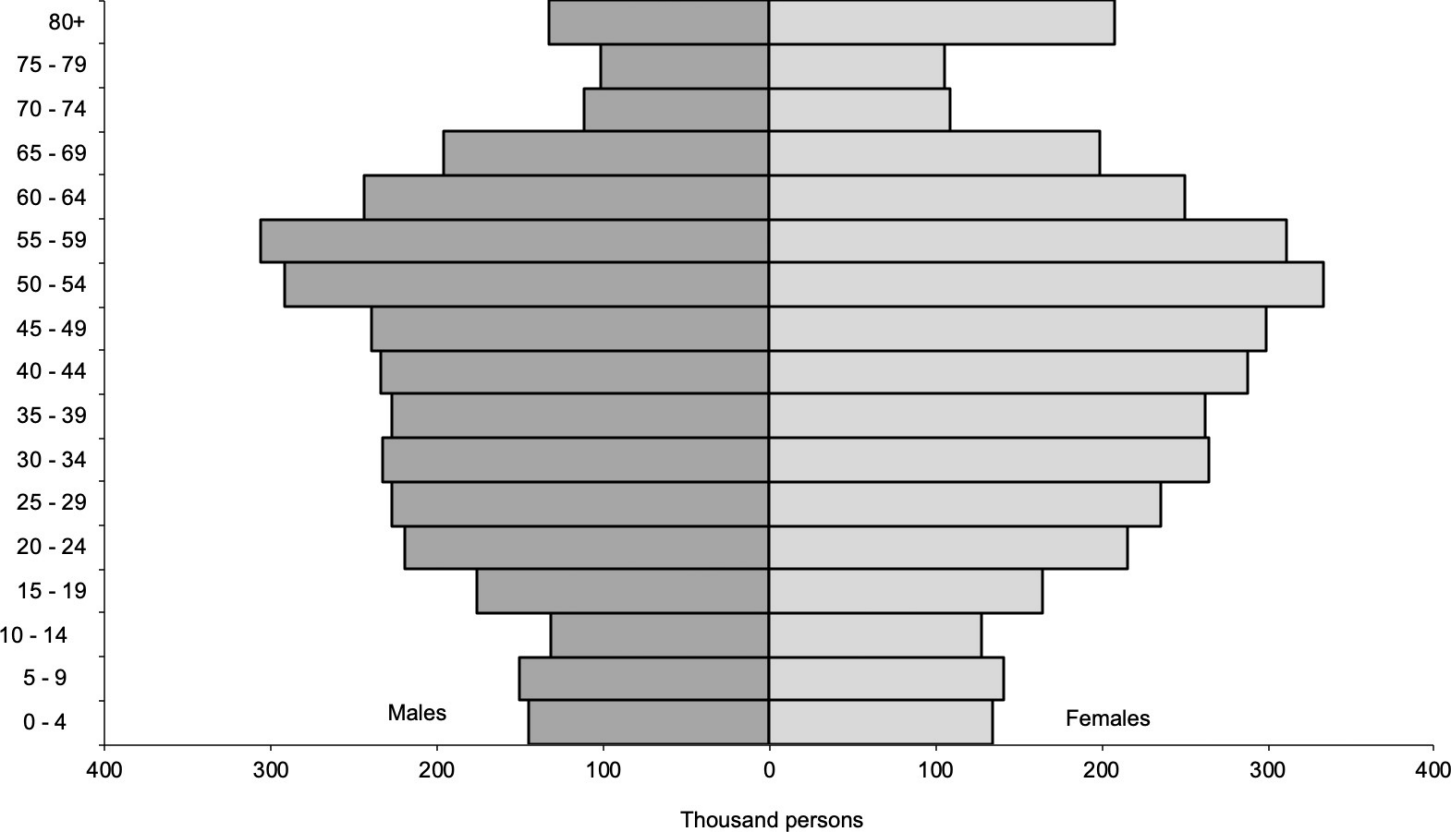
Population pyramid of Hong Kong, 2016. Data Source: 2016 Population By-census Office, Census and Statistics Department.

A recent paper highlighted the importance of a demographic approach to understanding COVID-19 transmission and fatality rates [18]. The paper suggested that ‘the age structure of a population may help explain differences in fatality rates across countries and how transmission unfolds.’ Fig 5 shows the full population pyramid for Hong Kong in 2016. Observing the discrepancy in the shapes of the pyramids in Figs 5 and 1, it is clear the experience from Hong Kong suggests that age structure alone is not a universal factor in shaping transmission and fatality rates. Despite having more than 18.0% of the population 65 and over and an extremely high population density, a package of policies designed to contain the virus spreading from younger imported cases and becoming a sustained local outbreak ensured that case and mortality numbers stayed low.

A further difference between Hong Kong and other settings characterized by higher rates of infection (and fatality) is the lower levels of distribution of residential care and support among older persons. There is strong evidence that people living in residential/nursing homes are particularly vulnerable to not only infection and its rapid spread, but to severe COVID-19 infection and fatality [19–21]; so much so that the WHO referred to care home infection and fatality rates as an “unimaginable human tragedy” [22]. In a study of official data in ten countries, deaths in care homes account for between 19–72% of all deaths [21] - although international comparison is difficult because of differences in cause of death reporting as testing procedures. In common with other parts of the world, the Hong Kong government has issued guidelines to support residential care homes in preventing infection [23], as well as offering other support in terms of provision of personal protective equipment (PPEs) and infection protection services [24] and a switch to online care support for those who would ordinarily visit day care centers [25]. There may thus be institutional reasons for the low levels of transmission among older people. The size and percentage of the older population resident in care homes differs widely around the world [26]. In the UK, where a high percentage of infections and fatalities have occurred in care homes [19,20], it is estimated that around 5.3% of the population aged over 70 is resident in care homes [27,28]. In addition, a further 6.9% of that population receive care support in their own homes, including from carers making multiple home visits in a day—potentially another area of risk for infection [27,28]. In contemporary Hong Kong, meanwhile, the proportion of over 70s in residential/nursing homes is estimated to be around 3.6% [1,29]. These lower rates of care home residence may have contributed to lower overall transmission and fatality numbers among the elderly by creating lower opportunities for sustained local spread within the elderly population.

Low-income migrant workers, an already neglected group in terms of health and other support mechanisms during this pandemic [30], represent a further group often characterized by communal living. Highly elevated transmission rates have been seen in some settings where such migrant workers often live in cramped, unsanitary conditions [31]. In Singapore, for example, over half of the purpose-built and factory-converted dormitories have been affected [32]; a factor held primarily responsible for the ‘second wave’ of infections in the city-state. It has been estimated that some 80% of all cases have been linked to such dormitories. Compared to Singapore, such ‘dormitories’ are rare in Hong Kong. Perhaps the primary, related housing issue in Hong Kong is 'subdivided housing’; home to up to 209,000 poor, urban individuals [33] and sometimes referred to as ‘coffin houses’ because of their very small sizes [34]. While often characterized by poor hygiene, low environmental [35] and safety standards [36], it appears that policy measures in Hong Kong, which succeeded in stemming local transmission chains, meant these quasi-communal units were not left exposed to rapid COVID-19 transmission.

A final possible factor mentioned by Dowd et al. [18] concerns the possibility that ‘intergenerational interactions, co-residence, and commuting may have accelerated the outbreak in Italy through social networks that increased the proximity of elderly to initial cases.’ This would be derived from a mechanism whereby the younger population most susceptible to initial infection [37] transmit to the elder population through such contact. In Hong Kong, multi-generational residence is common [38], where around 50% of those aged 65 and over live with their adult children [39] - much higher than in Italy (or, indeed, any other setting in Europe or North America characterized by high transmission rates) [40]. The extremely high population density of Hong Kong coupled with short distances and highly efficient transport systems means that there is a high degree of residential proximity as well regular contact between older parents and their children. A further dimension of intergenerational, intra-household interaction involves migrant domestic workers, who increasingly operate a major means of care support within the household. There is little evidence in Hong Kong that such workers were responsible for transmission within the household. As such, the hypothesis suggested for Italy appears inconsistent with the Hong Kong case at least.

The general quality of population health data in Hong Kong is generally accepted to be high, not least because of the centralized healthcare system in the territory. Furthermore, in this case a very high degree of health surveillance and monitoring was in force throughout the period. This analysis relied heavily on secondary data for both the information on the confirmed cases and travel histories. Given this, there are possible concerns for under diagnosis and under reporting especially during the start of the outbreak until before its first peak as have also been observed in other countries [41]. The unique age and sex distribution of the cases in Hong Kong may also be affected by testing patterns. There may, of course, have been unreported and/or asymptomatic cases in Hong Kong which will have escaped our analysis. However, there is no current consensus on such rates of asymptomatic infection and, therefore, how one might either estimate or correct in the Hong Kong case [42]. Finally, the challenge of ascertaining primary cause of death and the role played by COVID-19 interacting with other factors [43–45] is not a significant issue in Hong Kong given the very small number of deaths.

## Conclusion

A major limitation is that the spread of COVID-19 has not yet ended. Any observations of a ‘case in progress’ are prone to future, unexpected changes which will change the narrative already described. Of course, by the time this paper is published it is eminently possible that events have taken a turn for the worse and a *third* wave of infections occur. Despite this, Hong Kong has clearly demonstrated a capacity to control both the transmission and fatality of COVID-19 *until this point*. As of June 11, 2020 there have been no COVID-19 ascribed deaths since March 14, 2020. Furthermore, as of April 29, 2020, Hong Kong has reported no new cases for the sixth time in ten days [10], and there were just five cases of local transmission in the month of May. From the start of May, public facilities began to reopen, some border restrictions were lifted, and civil servants returned to work in their offices. As of mid-June, public facilities such as beaches, libraries, museums, swimming pools have largely reopened, as have the last group of commercial leisure/entertainment facilities (karaoke lounges, nightclubs, bathhouses and party venues).

Clearly, much more research is required to concretely establish both the epidemiological and social factors contributing to the Hong Kong experience. The data which will allow such analysis will only come on stream in the future. At this point more complex statistical analysis will be able to be performed. Furthermore, it will then become possible to link data on transmission and fatality through to other clinical and vital records (including the planned Census in 2021). Such data will also need to be complimented by survey data and qualitative research to provide a broader sense of the Hong Kong context.

Policy interventions, institutional systems, household and living arrangements each played a role in a complex, interwoven way. However, we must not overlook the role played by the community itself who, through behavioral change and increased vigilance, appear to have been equally instrumental in shaping Hong Kong’s COVID-19 experience. It may be in this way that the greatest lessons of SARS have been learned [5].

## Supporting information

S1 Table Timeline of major events and interventions related to the outbreak of the novel coronavirus (COVID-19) infection in Hong Kong. (DOCX)

S1 File Description of the data sources. (DOCX)
