# Supplementary material for: Exploring the young demographic profile of COVID-19 cases in Hong Kong: Evidence from migration and travel history data
Source: PLoS One. 2020 Jun 26;15(6):e0235306. doi: 10.1371/journal.pone.0235306 (PMC7319322; doi:10.1371/journal.pone.0235306)
Supplement: S1 Table — (DOCX) [file pone.0235306.s001.docx]

**Policy Measures**

We also gathered data on policy measures implemented by the Hong Kong government, highlighting the impact of major border closures and quarantine arrangements imposed by the Hong Kong government in reducing further numbers of imported COVID-19 infections. A timeline of major events and interventions is listed below.

**Timeline of major events and interventions related to the outbreak of the novel coronavirus (COVID-19) infection in Hong Kong**

| **Date** | **Events and Interventions** |
| --- | --- |
| January 23, 2020  January 24, 2020  January 25, 2020  January 27, 2020  January 30, 2020  January 31, 2020  February 04, 2020  February 05, 2020  February 08, 2020  February 13, 2020  February 25, 2020  March 1,2020  March 8, 2020  March 13, 2020  March 14, 2020  March 15, 2020  March 17, 2020  March 19, 2020  March 25, 2020  March 28, 2020  March 29, 2020 | Two important cases of novel coronavirus were confirmed. The Lady MacLehose Holiday Village under the Leisure and Cultural Services  Department (“LCSD”) was activated as a quarantine center  Flights and high-speed train services between Hong Kong and Wuhan were suspended  The Chinese New Year holidays of secondary schools, primary schools, kindergartens, child care centers, and special schools so that they would resume classes extended not earlier than February 17, 2020.  Immigration restrictions on Hubei Province residents and persons visited Hubei Province in the past 14 days  The World Health Organization (WHO) declared that the outbreak of the novel coronavirus constituted a Public Health Emergency of International Concern (“PHEIC”)  Classes in all schools in Hong Kong were suspended, with classes  to resume no earlier than March 2, 2020. Special work arrangement for government departments was extended until February 9, 2020, All-out efforts to support frontline healthcare staff and increasing supply of surgical masks through a multi-pronged approach were announced by the government.  First death confirmed.  Cross-boundary passengers by land or by sea converged to Shenzhen Bay control point and Hong Kong-Zhuhai-Macao Bridge (“HZMB”) Hong Kong Port (clearance service suspended at other four control points)  A 14-day compulsory home quarantine arrangement for all the people entering Hong Kong from Mainland China was announced.  Compulsory home quarantine of persons arriving from mainland China.  Special work arrangement for government departments was extended until February 23, 2020. The government announced that all schools in Hong Kong would not resume classes before March 16, 2020.  Non-Hong Kong residents arriving from South Korea are restricted from entering Hong Kong .  All arrivals (regardless of whether they are Hong Kong residents) to Hong Kong who have been to the Emilia-Romagna, Lombardy or Veneto regions in Italy or Iran in the past 14 days must stay in a quarantine centre for 14 days.  All inbound travelers arriving at the Hong Kong International Airport (HKIA) are required to complete and submit a health declaration form  Red OTA issued for the Schengen area, announcement of mandatory 14-day home quarantine for all arrivals from the Schengen area starting from March 17th.  Mandatory 14-day home quarantine for all people who have been to Italy (except Emilia-Romagna, Lombardy and Veneto regions), Bourgogne-Franche-Comte and Grand Est regions in France, the North Rhine Westphalia region in Germany, Hokkaido in Japan as well as the La Rioja, Madrid and Pais Vasco regions in Spain in the past 14 days, regardless of whether they are Hong Kong residents.  Red OTA issued for the United Kingdom and United States of American. Announcement of mandatory 14-day home quarantine for all arrivals starting from March 19th.  Mandatory 14-day home for all people who have visited the European Schengen Area* in the past 14 days regardless of whether they are Hong Kong residents.  Mandatory 14-day home for all overseas arrivals into Hong Kong regardless of whether they are Hong Kong residents.  Reject entry of non-residents from overseas (except citizens of mainland China, Macau S.A.R. and Taiwan).  Regulations on the operation of dining and other entertaining business.  Restriction on social distancing (prohibition of group gatherings with more than four people in public places). |
