# Supplementary material for: Exploring the young demographic profile of COVID-19 cases in Hong Kong: Evidence from migration and travel history data
Source: PLoS One. 2020 Jun 26;15(6):e0235306. doi: 10.1371/journal.pone.0235306 (PMC7319322; doi:10.1371/journal.pone.0235306)
Supplement: S1 File — (DOCX) [file pone.0235306.s002.docx]

**Description of data sources**

**Confirmed Cases Data**

Data related to confirmed COVID-19 cases was taken from the Centre for Health Protection [CHP] of the Hong Kong Department of Health. We assessed the age and sex distribution of the confirmed cases, discharge status (discharged, hospitalized or died), and type of transmission. We estimated the trend of cumulative cases by age group from January 23 to April 16, 2020, to determine the age group that registers the highest number of confirmed cases over time. See <https://www.coronavirus.gov.hk/eng/index.html>

**Population Data**

We utilized data for comparative demographic analyses from the Hong Kong Census and Statistics Department, United Nations Population Division and the Chinese Center for Disease Control and Prevention (see references in manuscript).

**Migrant Flows**

Data on daily migrant inflows and outflows were retrieved from the Hong Kong Immigration Department (www.immd.gov.hk), showing arrivals at each border checkpoint into Hong Kong broken down by citizenship status from January 24 to April 16.

**R Code**

## This code pulls daily data related to migrant inflows and outflows from the Hong Kong Immigration Department website and writes it into two data sets containing data on inflows and data on outflows

library(xml2)

library(purrr)

library(rvest)

library(magick)

library(dplyr)

library(tidyr)

#set start date and number of days, create blank tbl_df's for results

start.date=as.Date("2020/01/24")

days<-c(0:87)

all_arr<-tibble(Border=as.character(),Date=as.Date(start.date),ResidencyStatus=as.character(),Arrivals=as.character())

all_dep<-tibble(Border=as.character(),Date=as.Date(start.date),ResidencyStatus=as.character(),Departures=as.character())

for (i in days){

#set date and webpage

today<-start.date+i

web_date<-paste(substring(today,1,4),substring(today,6,7),substring(today,9,10),sep="")

webpage_url <- paste("https://www.immd.gov.hk/eng/stat_",web_date,".html",sep="")

webpage <- xml2::read_html(webpage_url)

#pull daily data from web, find data table

daily <- rvest::html_table(webpage,fill=TRUE)[[1]] %>%

tibble::as_tibble(.name_repair = "unique") # repair the repeated columns

#delete columns and rows

daily<- daily[6:20,1:9]

#reshape using dplyr and tidyr

daily_arr<- daily %>% transmute("HKRes"=X2,"Mainland"=X3,"Other"=X4,"Border"=X1,"Date"=today) %>% gather(key="ResidencyStatus",value="Arrivals",HKRes:Other)

daily_dep<-daily%>%transmute("HKRes"=X6,"Mainland"=X7,"Other"=X8,"Border"=X1,"Date"=today) %>% gather(key="ResidencyStatus",value="Departures",HKRes:Other)

#add daily data to results df

all_arr<-bind_rows(all_arr,daily_arr)

all_dep<-bind_rows(all_dep,daily_dep)

}

#remove commas and change numbers to numeric class

all_arr$Arrivals<-as.numeric(gsub("\\,", "", all_arr$Arrivals))

all_dep$Departures<-as.numeric(gsub("\\,", "", all_dep$Departures))

**Travel History Data**

We retrieved detailed archived datasets pertaining to the travel histories of confirmed COVID-19 cases which are updated on an almost daily basis by the Centre for Health Protection. Our process for obtaining the final travel history dataset was as follows:

- Using the Hong Kong Government Open Data centre at data.gov.hk we extracted all historical CSV files updated by the Centre for Health Protection between January to April 2020.
- We merged the files and excluded travel histories pertaining to domestic travel (labeled as buses, trains, and ferries).
- We excluded a small number of journeys pertaining to outbound flights from Hong Kong.
- We restricted the dataset to unique cases based on the COVID-19 case ID number, date, and flight ID.
- For case IDs who had logged multiple flights in the travel history database, we took the starting city of their journey. For example, a case who had logged two flights from Paris to Dubai and Dubai to Hong Kong was coded as Paris.
- Based on the starting travel destination we coded the starting country of travel.
- We matched the case IDs to the confirmed cases dataset which included details about age, sex, the onset of symptoms, and date of confirmed test.
